# How are woodworking and baking represented as we walk from the workshop to the kitchen? Virtual reality environment modulates the representation of corresponding actions

**DOI:** 10.3758/s13423-026-03001-7

**Published:** 2026-09-10

**Authors:** Leon O. H. Kroczek, Michael Roidl, Angelika Lingnau, Marius Zimmermann

**Affiliations:** 1 Department of Clinical Psychology and Psychotherapy, Institute of Psychology, University of Regensburg, Regensburg, Germany; 2 Chair of Cognitive Neuroscience, Institute of Psychology, University of Regensburg, Regensburg, Germany

**Keywords:** Action representation, Virtual reality, Scene/environment/context

## Abstract

The recognition of actions presented as static photographs has been suggested to be modulated by the scene context—or image background —in which an action is presented, particularly for actions that are hard to recognize. This suggests scene context as an important feature when retrieving action representations, and poses the question whether scene context directly modulates the representational space of actions. Therefore, in this preregistered virtual reality (VR) study, we investigated in a group of *N* = 24 healthy participants how scene context by means of the (virtual) environment affects the representation of actions that do or do not correspond to the current environment. Participants performed multi-arrangement tasks on a set of kitchen and workshop actions while the virtual environment was manipulated to represent either a kitchen, workshop or neutral environment. Representational structure was estimated from the multi-arrangement tasks separately for each virtual environment and action domain (kitchen, workshop). Results showed increased consistency of the representation of kitchen actions over participants when the task was performed in a congruent (i.e., kitchen) compared with an incongruent (i.e., workshop) or neutral virtual environment, however, the same effect was not observed for workshop actions. Representational structure, however, was not affected by the environment. In accordance with previous studies on representational changes in domain-specific experts within various action and object domains the results suggest that an agent’s environment may modulate the action space for corresponding actions by increasing robustness of represented familiar actions. These findings contribute to our understanding of contextual effects on mental representations within the domain of actions and beyond.

**Supplementary Information:**

The online version contains supplementary material available at https://doi.org/10.3758/s13423-026-03001-7.

## Introduction

Actions of a particular category are often associated with specific environments: food is prepared in kitchens, teaching is done in classrooms, and furniture is constructed in workshops. Accordingly, previous studies have shown that an incongruent scene context interferes with action recognition (Wurm & Schubotz, 2012), and that compatible contexts facilitate the recognition of actions that are hard to recognize (Wurm & Schubotz, 2017). In these studies, the compatibility between actions and scene context was manipulated by static images depicting actions presented in a random order, with the scene context depicted in the background. By contrast, in everyday life, we observe actions belonging to related events (e.g., preparing a meal) unfolding within our environment, and we are assumed to have access to scripts regarding likely sequences of actions (Zacks & Swallow, 2007). Accordingly, the scene context (e.g., a kitchen) is already available before an action is observed and may therefore exert state-like effects on the processing and representation of actions observed within that context. This raises the question of whether and how the representational space of actions is modified when participants are exposed to a specific scene context by means of virtual reality.

A growing number of studies suggest that actions are represented in multidimensional action spaces where similar actions are located close to each other (Kabulska & Lingnau, 2023; Lingnau & Downing, 2024; Thornton & Tamir, 2022; Tucciarelli et al., 2019; Zhuang & Lingnau, 2022) and form clusters corresponding to superordinate action categories (Zhuang & Lingnau, 2022). Previous studies focussed on static aspects of action spaces; however, both experimental and theoretical accounts suggest that action spaces might in fact be modulated at varying timescales (e.g., via category-based attention to actions; Danaj et al., 2026; Lingnau & Downing, 2024; see also Gärdenfors, 2004) or long-term experience within particular action domains (Schack, 2004; Schack & Mechsner, 2006). As such, representations of actions are considered to follow similar principles as representations of objects (Hebart et al., 2020; Rosch et al., 1976). Specifically, the representational space of objects has been shown to be modulated by explicit shifts in attention (Chapman & Störmer, 2024; Çukur et al., 2013; Shahdloo et al., 2022; see also Nastase et al., 2017, for modulations of semantic feature representations), and by long-term expertise (Duyck et al., 2021; Martens et al., 2018). Moreover, context effects have been described for the recognition and decoding accuracy of objects both at the picture level (e.g., Brandman & Peelen, 2017; Davenport & Potter, 2004; Leticevscaia et al., 2024; see also Bar, 2004) as well as within augmented reality enhanced real-life environments (Nicholls et al., 2025). Taken together, these studies suggest extensive interactions between processing of different visually perceived entities.

Short-term, state-like changes in scene context, for example those elicited by moving from one environment to another (e.g., from the kitchen to the classroom), might thus affect the representational space of actions. Here, two potential effects will be considered. First, the action space might be reshaped—in particular, compressed or extended along one or more dimensions—thereby increasing sensitivity among one group of actions at the cost of sensitivity between other actions, comparable to attention-driven effects on action and object spaces (Chapman & Störmer, 2024; Lingnau & Downing, 2024; Shahdloo et al., 2022). Second, the representation of context relevant actions within the action space might become more robust to individual differences between action exemplars, possibly due to increased attention to relevant action features. Long-term expertise within a given (object) domain has been suggested to affect object representational spaces in similar ways, driven by intensive training and conceptual knowledge that is shared among experts (Duyck et al., 2021; Martens et al., 2018). Both mutually not exclusive processes would tune the agent towards facilitated processing of actions that are likely to occur within their surroundings.

In this preregistered study, participants performed multi-arrangement tasks on a set of kitchen and workshop actions while being situated in virtual environments representing kitchens, workshops or neutral rooms. Temporary modulations of action spaces as measured by modulated pairwise dissimilarities and increased within-group consistency of representational dissimilarity matrices were expected benefiting the subset of actions that corresponds to the (virtual) environment. Whereas previous studies focussed either on static aspects of representational spaces of actions, or on stimuli specific scene context effects on action recognition, here we focussed on effects of the scene context on the representation of actions. Specifically, we investigated whether scene context has temporary, state-like effects on these representations. To this aim, we made use of virtual reality (VR) to manipulate the scene context. Contrary to conventional screen-based paradigms, VR allows to present stimuli embedded within immersive, interactive 3D environments while maintaining a high level of experimental control (Bohil et al., 2011). Consequently, VR can be used to study behaviour and cognition in naturalistic contexts close to the real world (Schöne et al., 2023).

## Methods

### Preregistration and open science

This study was preregistered at OSF (https://osf.io/cze2h). The preregistered procedures were followed without changes. When additional (not preregistered) analyses were performed, this is indicated below. Specifically, we added a comparison of between-environment correlations with within-environment correlations as an investigation of structural similarity, based on previous work by Duyck et al. (2021) and Op de Beeck et al. (2008). We also added additional measures pertaining to changes in the representational space as described below.

All scripts used for data processing and analysis, as well as the raw and preprocessed data, excluding virtual reality scenarios (due to licensing limitations) are provided via OSF (https://osf.io/my92n).

### Participants

Twenty-four healthy participants (16 women, eight men) aged 20–33 years (mean ± *SD* = 24.0 ± 3.16) took part in the study for course credit. Sample size was determined based on previous studies comparing object representations in experts versus nonexperts using between-subject designs (Duyck et al., 2021). Participants were recruited at the University of Regensburg. All participants provided written informed consent for inclusion in the study before participation. The study was approved by the local ethics committee (project code 19–1510-101) and conducted in accordance with the Declaration of Helsinki.

### Stimuli

**Fig. 1:**
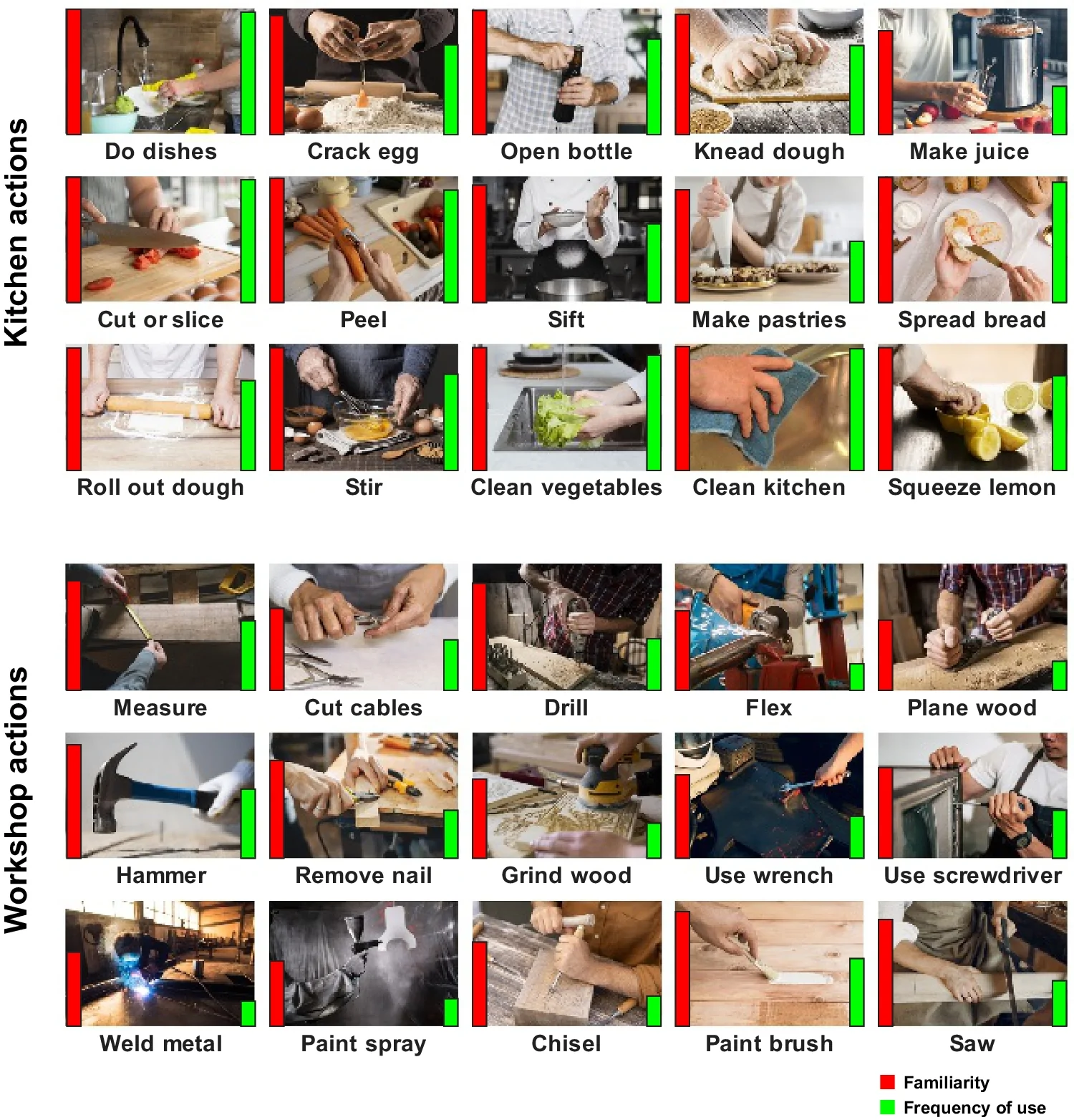
Actions stimuli and ratings. Set of kitchen (Rows 1–3) and workshop (Rows 4–6) actions and descriptive labels (labels were not provided to participants). For each action, one exemplar is shown. Red bars (left) indicate average familiarity, green bars (right) indicate average frequency of use, as rated by an independent group of participants on a 5-point Likert scale (see Supplementary material S1 for details). (Colour figure online)

For each action domain (kitchen actions, workshop actions), 15 representative actions were selected. To obtain the list of actions, first, we combined actions included in the work by Wurm and Schubotz (2012) with an unspecified number of actions created in ChatGPT (using prompts: *provide a list of typical actions that are performed in a kitchen*; *provide a list of typical workshop actions*; GPT-3.5; OpenAI, San Francisco, CA, USA). From these action lists, the authors (M.R. and M.Z.) selected actions that they subjectively deemed both plausible and representative for the respective context, and that could likely be recognized from static images. The final list of actions is presented in Fig. 1, along with scores for familiarity (i.e., knowing the purpose of the action) and frequency of use for each action, rated by an independent group of 50 bachelor students in psychology from the same university after data analysis of the main study (see Supplementary Material S1 for a full report). For each action, three exemplar pictures were collected from a license free image database (freepik). For each image, selection criteria were that (1) the content of the image is focused on the action, (2) the action is (spatially) presented within the central portion of the image, (3) the background is visible/not blurred, natural and action-congruent, and (4) pictures are in landscape format. Example images for all actions are shown in Fig. 1.

### Virtual environments

**Fig. 2:**
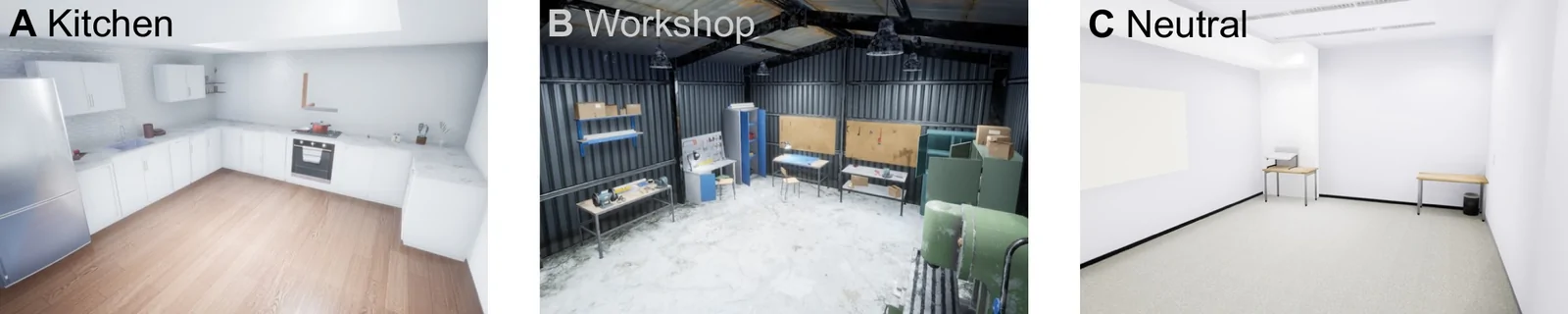
Virtual reality environments. Each image shows a screenshot of the virtual environments: **A** Kitchen, **B** Workshop, **C** neutral, empty room. In the lab environment, a table, where participants performed the multi-arrangement task, was placed in the centre of the virtual room (cf. Figure 3 A). (Colour figure online)

Three virtual environment rooms were used in the current experiment: a kitchen, a workshop, and a neutral (empty) room (Fig. 2). For the kitchen, the “Modular Kitchen” by “AndresAlv” (https://www.fab.com/listings/6f2d572d-cc0b-445d-a7a6-38c43c79398f) was used; for the workshop, we used the “Survival Workshop Garage” by “Tirgames Assets” (https://www.fab.com/listings/25def53d-f463-4b54-be8a-11e5ade3bac8). Both virtual rooms were equipped with virtual furniture and context specific objects and decorations from the respective packages and modelled to fit the local lab setup (see below). For the neutral condition, an in-house empty virtual room was used, which was dimensioned after common offices at the University of Regensburg.

The virtual environments were presented in 3D via a CAVE system (Cave automatic virtual environment) with a size of 3.6 m × 2.4 m × 2.5 m (L × W × H) and five projection planes surrounding the participant (front, left, right, back, and floor, BARCO F50 WQX6A projectors with a resolution of 2560 by 1600 pixels; Barco, Karlsruhe, Germany). Participants wore 3D shutter glasses with attached motion tracker targets (Advance Realtime Tracking GmbH, Weilheim in Oberbayern, Germany). VR was rendered using the Unreal 4 game engine (Version 4.27, Epic Games Inc., Cary, NC, USA) in a cluster of ten computers (i7–4790 k, GeForce 1080, 16 GB RAM).

### Multi-arrangement task and procedures

**Fig. 3:**
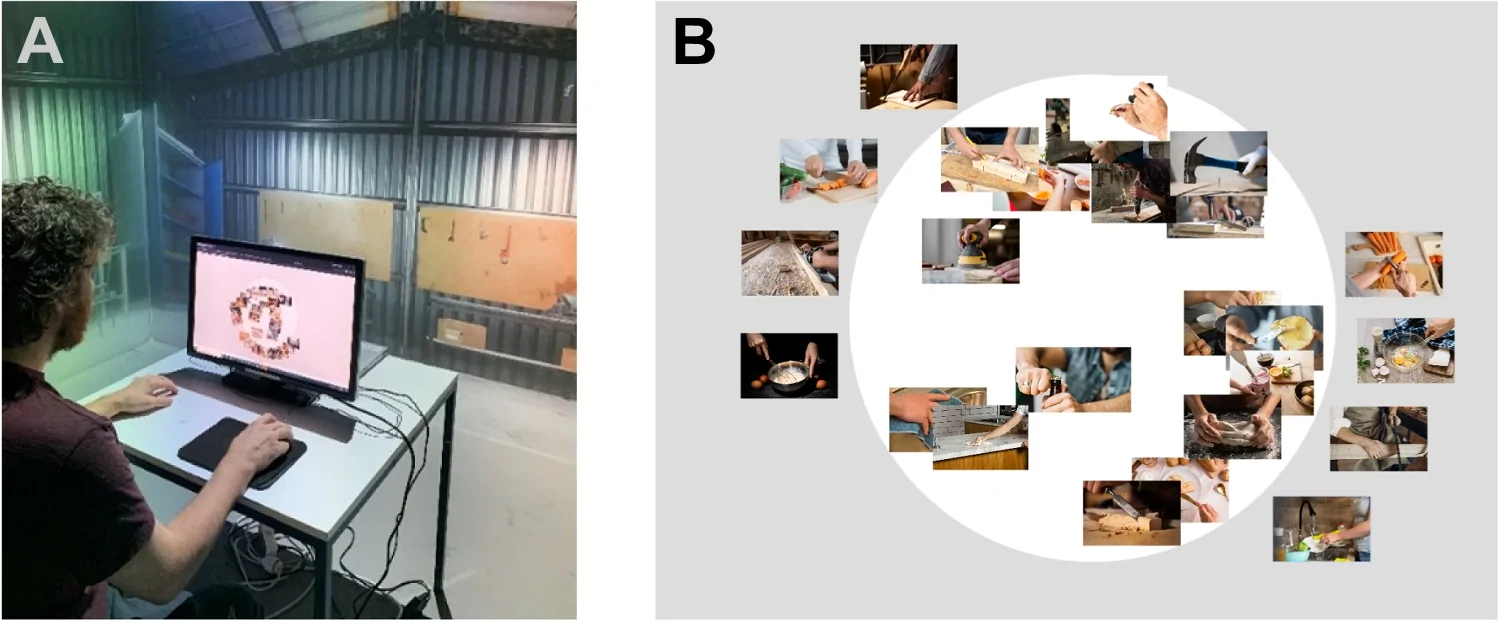
Virtual reality lab setup and multi-arrangement task. **A** The physical setup within the virtual reality lab (CAVE System). Participants were seated at a table in the centre of the virtual reality room (here: the workshop; see Fig. 2 for all examples), performing a multi arrangement task on a computer screen. Participants wore 3D shutter glasses with motion tracking allowing to render the virtual room in accordance with the participants’ position and head direction during the entire time inside the CAVE environment (not shown here). Note that the picture of the CAVE environment only shows a 2D reproduction of the 3D environment that was presented to the participants via shutter glasses. **B** Detail of the screen setup during the multi arrangement task, using the meadows online platform (meadows-research.com). (Colour figure online)

Participants were asked to perform a multi-arrangement task (Kriegeskorte & Mur, 2012) with two sets of stimuli, kitchen actions and workshop actions (see Stimuli; Fig. 1), in three virtual environments: a kitchen, a workshop, and a neutral (empty) room (see Virtual Environments; Fig. 2). In this task, participants were asked to arrange images depicting actions by their perceived similarity in a (virtual) arena. No specific instructions were given regarding the criterion used for the arrangement, following the procedures of previous studies (e.g., Kriegeskorte & Mur, 2012). The task was presented on a 22-inch monitor which was physically located on a table in the centre of the CAVE system (1,680 × 1,050 px, 60 Hz; distance approx. 40 cm; Fig. 3A).

Before starting the multi-arrangement task, participants were given two minutes to familiarize themselves with the virtual room and its interior while roaming around freely in the virtual room. Instructions were given through prerecorded spoken instructions, including a prerecorded 2-min silent period, and concluded with the instruction to initiate the task. The instruction was as follows:

“*Please take a moment to familiarise yourself with the entire virtual room and try to memorise its details. After the sorting task that follows, there may be a questionnaire asking about details of the room.* [2 minutes silence] *You may now sit down at the table and begin the sorting task.*”

The multi-arrangement task followed the procedures outlined by Kriegeskorte and Mur (2012) and was administered in Meadows (meadows-research.com). During the first trial, the full set of basic level actions, 15 kitchen and 15 workshop actions (see Fig. 1 for a full list), were presented in a circular arrangement on the screen. For each action, one of the three exemplars was chosen at random per virtual context, without replacement (i.e., exemplars were not repeated in different virtual contexts). Participants were asked to arrange the action images by drag and drop using a computer mouse (Fig. 3). Once they completed the initial arrangement, they could advance to the next trial. In each following trial, a subset of the stimuli was selected using an adaptive algorithm that aims to optimize evidence for dissimilarity estimates, until sufficient evidence was gathered (see Kriegeskorte & Mur, 2012, for details; evidence utility exponent = 10, min. required evidence weight = 0.5). At this point, the participant was informed that the task was completed, and participants were guided out of the VR environment. Outside, participants were asked to complete a memory task and fill in an immersion questionnaire (see below). This procedure was completed three times, until the task was performed in all virtual contexts. The order of virtual environments was counterbalanced across participants. The total experiment lasted approx. 60 min.

### Questionnaires

Following each VR run, participants were asked to complete a short memory task in which they were presented with a list of five (German) object names (*kitchen environment:* apple, cutting board, window, pot, salad bowl; *workshop environment:* cardboard, ladder, vacuum cleaner, paint bucket, hammer), and asked to indicate whether these items were present or absent in the VR environment (i.e., within the modelled environment, *not* in the stimulus set presented during the multi-arrangement task). This task was intended to draw the participants’ attention towards the virtual environment. No memory task was completed following the neutral VR environment given that no objects besides a table were present in the room (see Fig. 2C), and the room acted as a baseline.

Additionally, participants completed the *physical presence* subdomain of the multimodal presence scale (MPS; Makransky et al., 2017) to measure their subjective feeling of presence for each virtual environment. A one-way analysis of variance (ANOVA) indicated significant differences between virtual environments, *F*(1.59, 36.51) = 4.37, *p* =.027, η_p_^2^ =.160; Greenhouse–Geisser corrected); however, post hoc comparisons using a Tukey HSD test did not reveal significant pairwise differences between kitchen (13.63 ± 4.52), workshop (15.04 ± 4.19) and neutral environment (12.83 ± 4.56; all *p* values >.05). At the end of the experiment, participants were asked to indicate physical sickness symptoms using the simulator sickness questionnaire (Kennedy et al., 1993). None of the participants indicated increased simulator sickness (range: 1–15; mean ± *SD*: 5.5 ± 3.74; threshold: 20; Stanney et al., 1997).

### Planned analyses

For each run of the multi-arrangement task, we estimated the representational dissimilarity matrix (RDM) including all 30 basic level actions (15 kitchen and 15 workshop actions) using inverse multidimensional scaling (Kriegeskorte & Mur, 2012) and then divided them by action domain. Accordingly, we obtained six RDMs per participant, for each combination of virtual environment and action domain.

First, we computed the average distance per participant between action pairs for each of the six RDMs, providing a general measure for the dissimilarity between all actions within an action-domain during a given virtual context. These average distances were analysed using a two-way repeated-measure ANOVA with factors action domain (kitchen actions, workshop actions) and virtual environment (VR kitchen, VR workshop, VR neutral).

Next, we computed leave-one-out correlations for each combination of virtual environment and action domain as a measure of consistency (cf. Duyck et al., 2021; Op de Beeck et al., 2008). Specifically, for each combination of action domain and virtual environment, the upper triangle of the RDM for each individual participant was correlated with the upper triangle of the average RDM over all other participants, using Spearman rank correlations. These correlation values were Fisher *z* transformed and analysed using a two-way repeated-measure ANOVA with factors action domain (kitchen actions, workshop actions) and virtual environment (VR kitchen, VR workshop, VR neutral). Additionally, we computed between-environment leave-one-participant-out correlations between virtual environment pairs (kitchen–workshop, kitchen–neutral, workshop–neutral), and compared these, using one-sample *t* tests, to a reference value corresponding to the mean between the corresponding within-environment leave-one-participant-out correlations (Duyck et al., 2021; not preregistered). This comparison reveals significant differences in the similarity structure given that the within-environment correlations provide a ceiling level for between-environment correlations (Op de Beeck et al., 2008).

For each run, the number of correctly remembered items was counted and compared using one-way repeated-measure ANOVAs. Moreover, we measured subjective immersion with each virtual environment using the *physical presence* subdimension of the multimodal presence scale (Makransky et al., 2017).

All analyses were performed in MATLAB 2025a (The MathWorks, Natick, MA, USA). Alpha level was set to *p* <.05. Significant effects were followed up with post hoc multiple comparisons using the Tukey HSD test. Mauchly’s Tests of Sphericity indicated no violations of sphericity for the RDM measures (correlation and distance; all *p* values >.10), but for the MPS test scores (*p* =.036; accordingly, Greenhouse–Geisser correction was applied in the comparison). One-sample Kolmogorov–Smirnov tests indicated no violation of normality for any of the RDM measures (correlation and distance) or MPS scores (all *p* values >.10). A sensitivity analysis (G*Power 3.1.9.6; *F* test with repeated measures, within factors; Faul et al., 2007) indicated that the study design and sample size had 80% power to detect effects of virtual environment on representational consistency of at least *f* =.25 (95% power to detect effects of at least *f* =.32), and on average representational distance of at least *f* =.21 (95% for *f* =.26), suggesting that moderate effects could reliably be detected.

## Results

### Expansion and compression of representational space

**Fig. 4:**
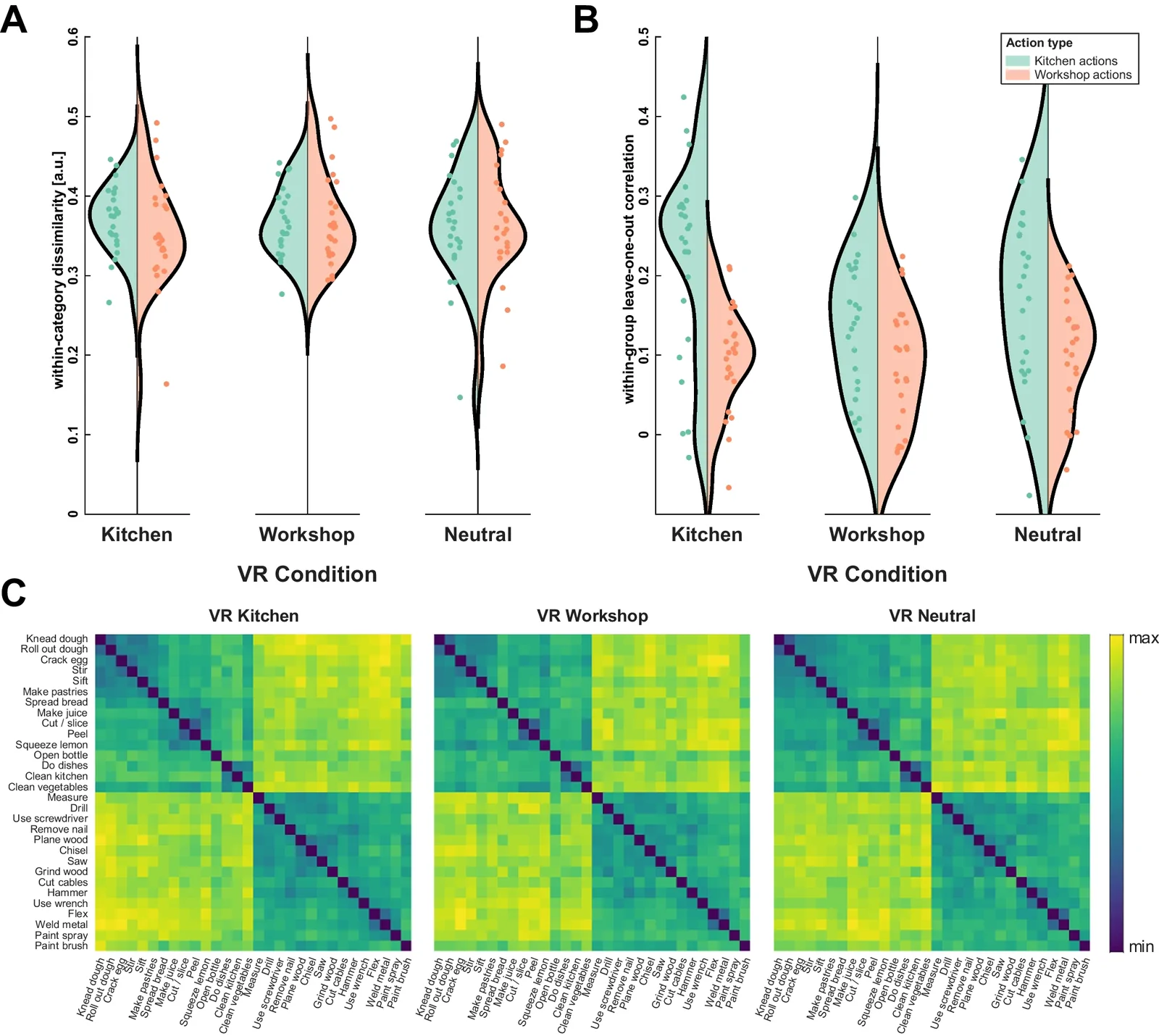
Representational similarity and consistency and dissimilarity matrices. **A** Average dissimilarity between action pairs by virtual environment and action domain. **B** Within-group consistency of representational dissimilarity. Raincloud plots created using Allen et al. (2021). Dots represent individual data points. **C** Group average representational dissimilarity matrices by virtual environment. Order of actions is based on agglomerative hierarchal clustering in the neutral VR condition, using the *average* method for computing distances between clusters. (Colour figure online)

We computed the average (not normalized) distance between all action pairs, separately for kitchen and workshop actions, in each virtual environment (Fig. 4A). A two-way repeated-measures ANOVA indicated no significant main effects of action domain, *F*(1,23) = 0.26, *p* =.615, η_p_^2^ =.011, and virtual environment, *F*(2,46) = 0.68, *p* =.511, η_p_^2^ = 0.029, and no interaction, *F*(2,46) = 1.24, *p* =.298, η_p_^2^ =.051. This indicates no evidence for a modulation of the action space in terms of expansion or compression.

Given that there are currently no standards for analyses quantifying the expansion and compression of representational spaces, we repeated the analysis with other measures (not preregistered), including average distance following nonclassical two-dimensional scaling of the dissimilarity scores (using mdscale, Euclidean distance) and cluster extent (avg. distance of all items to centroid) in kmeans clustering within a single cluster per action type. None of these measures resulted in significant interaction effects, further indicating no evidence for a modulation of the action space as a function of virtual environment.

### Consistency

To estimate consistency of representational spaces over participants, we computed leave-one-out correlations between participants for each action domain and virtual environment (Fig. 4B). A two-way repeated-measures ANOVA indicated a significant interaction between action domain and virtual environment, *F*(2,46) = 5.61, *p* =.007, η_p_^2^ =.196. Moreover, the ANOVA revealed a significant main effect of both action domain, *F*(1,23) = 38.68, *p* <.001, η_p_^2^ =.627, and virtual environment, *F*(2,46) = 6.24, *p* =.004, η_p_^2^ =.213.

Post hoc comparisons using the Tukey HSD test indicated that kitchen actions were arranged significantly more consistent across participants than workshop actions (within kitchen environment: *p* <.001; within workshop environment: *p* =.049; within neutral environment: *p* =.006), in line with the main effect of action domain. Further post hoc comparisons using the Tukey HSD test indicated that the consistency difference between kitchen and workshop actions was larger in the kitchen environment than the workshop environment (*p* <.001) and the neutral environment (*p* =.001), and smaller in the workshop environment than the neutral environment (*p* =.002). Accordingly, kitchen actions were significantly affected by virtual environment, *F*(2,46) = 7.83, *p* =.001, η_p_^2^ =.254, whereas workshop actions were not significantly affected by virtual environment, *F*(2,46) = 0.56, *p* =.576, η_p_^2^ =.024. This indicates increased consistency over participants for kitchen actions within the kitchen environment compared to workshop and neutral environments.

Next, we calculated pairwise between-environment correlations (not preregistered; cf. Duyck et al., 2021) and compared these to within-environment correlations. Significantly lower between-environment correlations compared to a reference value that lies in between the corresponding within-environment correlations (i.e., consistency measures; computed as the square root of the product of the respective within-environment correlations) would indicate that the representational structures differ between the respective environments (see Duyck et al., 2021; Op de Beeck et al., 2008). The between-environment correlations for all environment pairs (kitchen–workshop, kitchen–neutral, workshop–neutral) did not differ significantly from the respective reference values (all *p* values >.10), indicating that the representational structure of both kitchen and workshop actions was qualitatively similar in all virtual environments.

### Processing of virtual environments

Processing of virtual environments was tested in a memory task probing participants’ memory for items presented in each scene. Accuracy was higher for the kitchen environment (95.0 ± 8.66%; mean ± *SD*) compared to the workshop environment (78.3 ± 15.18%), *F*(1,23) = 22.12, *p* <.001. It should be noted that this difference might be due to different number of objects within the VR environments, which was larger for the workshop environment.

## Discussion

In this study we investigated how action representations are affected by the environment in which an agent is situated. In contrast to previous studies investigating context effects at the picture level (Wurm & Schubotz, 2012, 2017), here we manipulated the actual (albeit virtual) environment, rather than the background of an action image itself. We made use of VR to create the impression of being in different environments. We observed that the virtual environment does affect the consistency of the representational space of actions across participants in some, but not in other environments. Specifically, the representation of kitchen actions was affected by the presence in a (virtual) kitchen compared with a different (neutral or incongruent) environment, however, the same was not the case for the representation of workshop actions. At the same time, we did not observe structural changes in representational spaces in response to the environment.

Previous studies investigating the role of scene context on the perception of actions focussed on recognition performance, for example, by manipulating whether an action is presented within a congruent or incongruent scene (Wurm & Schubotz, 2012, 2017). These studies have shown that the (stimulus specific) scene context directly influences, at least in ambiguous situations, how fast and accurately individual actions are perceived. Other studies have shown that scene features, such as whether an action typically takes place outdoors, are associated with specific action categories and explain variability in the category-based structure of action categories (Kabulska & Lingnau, 2023). Moreover, it has been shown that contextual scene features are represented in the cerebral cortex during action recognition (Kabulska et al., 2024; Zimmermann & Lingnau, 2025). Finally, in data driven approaches the environment (nature/outdoor) has been proposed to form a distinct dimension within a presumed multidimensional space in which actions are represented (Bockes et al., 2025; Dima et al., 2023). Taken together, these studies suggest that actions are not recognized, and likely represented, in isolation, but in interaction with the environment in which they take place. That said, previous studies investigating representational spaces of actions focused on static aspects of these spaces (Bockes et al., 2025; Dima et al., 2023; Kabulska & Lingnau, 2023; Thornton & Tamir, 2022; Tucciarelli et al., 2019). The current study went one step further, by manipulating the environmental context in which an agent is situated while estimating the representational space of actions. The idea behind this manipulation is that, in everyday life, we are aware of our own environment which only changes at a slow rate—for example, as we move from one room to another—and we perceive actions within this environment. Under the assumption that the representational space of actions can be modulated by experience, attention and possibly other factors (Lingnau & Downing, 2024), we would expect a shift in representational space through the manipulation of an agent’s environment. We observed such a change when participants were situated in a virtual kitchen with respect to representation of kitchen actions, however, we did not observe the same effect with representations of workshop actions in a virtual workshop environment.

Whereas we did not directly observe a change in representational structure between virtual environments, such as a zooming or distortion of representational space (cf. Chapman & Störmer, 2024; Lingnau & Downing, 2024), we did observe an increase in interindividual consistency of the representational space of kitchen actions when processed in a virtual kitchen environment. Specifically, the representational space of kitchen actions of independent individuals became more alike when these were situated in virtual kitchens, compared with when they were situated in virtual workshops or neutral rooms. Interindividual alignment of mental representational spaces has previously been observed within various expert groups, specifically, in ornithologists with respect to the representation of birds and minerals in respective experts (Duyck et al., 2021; Martens et al., 2018), and expert tennis players with respect to tennis serves (Schack, 2004; Schack & Mechsner, 2006) and ballet dancers (Bläsing & Schack, 2012). In these studies, long term experience likely shaped the representational space specific to a trained expertise—in our study, short-term changes in the (virtual) environment had comparable consequences. However, why would the representational space of ‘kitchen actions’ be affected by entering a kitchen, but not the representational space of ‘workshop actions’ by entering a workshop? We do not suggest that moving to a different environment makes you an expert for this environment—however, given that the population from which our participants were recruited (Western, educated, industrialized, rich, and democratic) presumably is fairly familiar with most kitchen actions that were presented in the current study (cf. Figure 1 for ratings of familiarity and frequency of use of each of these actions, obtained from a comparable participant group, supporting this post hoc explanation), it should be safe to suggest that our participants are “kitchen experts” to some extent. By “entering” a kitchen, then, this expertise dynamically modulated the representational space of kitchen actions into a more structured and robust action space, where representations of kitchen actions are more invariant to individual differences between action exemplars. We speculate that these temporary modulations of the representational space are due to increased attention towards critical action features that are shared between actions of the same type, thereby reducing variance between concrete instances of these actions. Consequently, each action within this space could be located more precisely, resulting in interindividually more similar action spaces (see also Duyck et al., 2021). Workshop actions, in contrast, might not be represented to the same extent and detail as kitchen actions due to differences in the exposure to and experience with kitchen and workshop actions, reducing or eliminating this effect. This explanation would fit with previous observations of domain-specific expertise effects in object representation (Martens et al., 2018). However, differences in terms of familiarity and frequency of use between actions belonging to different action domains might have limited their comparability. Future studies could instead include actions and environments from two equally familiar domains. Moreover, since participants’ experience was not assessed individually, this interpretation must be treated with care, and future studies are required that further investigate this hypothesis. Additionally, it needs to be shown that different exemplars of the same action are placed more consistently also within participants by including multiple varying exemplars per action within the same (virtual) environment.

Regarding the lack of measurable changes in representational structure between virtual environments, it has been suggested that attention and experience, or other factors, can distort the representational space through expansion or compression of specific areas within the space (Chapman & Störmer, 2024; Lingnau & Downing, 2024). Accordingly, for the current study, we expected that the representational space of actions from a given domain would be expanded in the corresponding environment, and/or that the representational space of actions would be compressed in the incongruent environment. However, neither effect was observed in our data, despite the change in interindividual consistency discussed above—suggesting that the overall structure of the representational space remained unchanged. This interpretation is further supported by the comparison of between-context correlations with reference values based on the within-context correlations, indicating that actions are arranged in a similar manner in all contexts (see also Duyck et al., 2021).

Taken together, here we observed that, in some situations, a change in (virtual) environment affects the interindividual consistency of the representational space of actions that fit within the environment, but not in others. These changes reflect what has been observed in experts with domain-specific knowledge regarding objects or actions (Bläsing & Schack, 2012; Calvo-Merino et al., 2005; Collins & Behrmann, 2020; Duyck et al., 2021; Martens et al., 2018; Schack, 2004; Schack & Mechsner, 2006), and suggest that the representational space of corresponding actions becomes more robust, provided an individual is knowledgeable about an action domain. To confirm our interpretation that changes in the environment affect the representational space of familiar actions, future studies should investigate both, additional environments and action domains, as well as levels of domain-knowledge. Would we, for example, see an effect on workshop actions when including a group of carpenters rather than predominantly students of psychology? And do the differences between experience scale from novices to hobby-users to professional users?

Future work should continue exploiting and discussing the benefits of immersive VR, because it allows for controlled and systematic manipulations of environmental and action-related features. This makes it possible to examine how environmental context shapes action representations in a detailed and ecologically valid way that traditional laboratory-based approaches cannot achieve.

### Conclusion

By exploiting the benefits of VR, we provided evidence suggesting that changing the environment in which we are situated may modulate the representational space of corresponding actions if a person is familiar with the action domain. Actions that are likely to occur within an environment are represented more consistent over individuals, indicating increased robustness of these representations. These results extend previous findings on scene-action compatibility using conventional screen-based paradigms, which showed facilitated recognition of actions in compatible scenes. Using an immersive VR paradigm that places participants in a 3D scene, our work suggests that scene context not only facilitates action recognition but may be exploited to actively prepare the brain for upcoming events, in particular observed actions, as we explore our spatial environment. These findings could also broaden our understanding of the mental representations of actions, suggesting that representations of actions are not static entities, but subject to temporary changes governed by our environment and possibly additional variable properties.

## Supplementary Information

Below is the link to the electronic supplementary material.

Supplementary file1 (DOCX 102 kb)

## Data Availability

The data for the experiment are available at OSF: https://osf.io/my92n.

## Code availability

The code for the experiment is available at OSF: https://osf.io/my92n.

## Declarations

### Open practices statement

The data and materials for the experiment are available at OSF (https://osf.io/my92n). The study was preregistered (https://osf.io/cze2h).
