# Supplementary material for: How are woodworking and baking represented as we walk from the workshop to the kitchen? Virtual reality environment modulates the representation of corresponding actions
Source: Psychon Bull Rev. 2026 Sep 10;33(7):235. doi: 10.3758/s13423-026-03001-7 (PMC13562166; doi:10.3758/s13423-026-03001-7)
Supplement: Supplementary file 1 [file 13423_2026_3001_MOESM1_ESM.docx]

**Supplementary material**

How are woodworking and baking represented as we walk from the workshop to the kitchen? Virtual Reality environment modulates the representation of corresponding actions

**Authors:** LOH Kroczek^1^, M Roidl^2^, A Lingnau^2^, M Zimmermann^2^

**Affiliations:**

^1^ Department of Clinical Psychology and Psychotherapy, Institute of Psychology, University of Regensburg, Germany

^2^ Chair of Cognitive Neuroscience, Institute of Psychology, University of Regensburg, Regensburg, Germany

**Supplementary material S1
Familiarity and frequency of use of kitchen and workshop actions in a sample of bachelor psychology students**

**Methods**

*Participants.* A new sample of 50 participants (44 female, 5 male, 1 diverse; 21.92 +- 3.92 years) was recruited among bachelor psychology students at the University of Regensburg using an online research platform. All participants had to be currently written in as bachelor student in Psychology. All participants provided online informed consent before participation. Participants were compensated with course credit.

*Task.* Participants were asked to rate each action, consisting of the same 15 kitchen and 15 workshop actions that were used in the main experiment, in terms of familiarity and frequency of use. For each action, one of the three exemplars was chosen at random, and presented in random order. Below the image, participants were asked to indicate how familiar they are with the action (i.e. knowing what is done in the action and what its purpose is), and how often they perform the action, using a 5-point Likert scale each. The task was self-paced and took approximately 10 minutes.

*Analysis.* We compared familiarity and frequency ratings using Wilcoxon rank sum tests, respectively, given that not all data came from a normal distribution, as established using one-sample Kolmogorov-Smirnov tests. Alpha level was set to .05 for both tests.

**Results**

Participants scored higher on both familiarity and frequency of use with respect to kitchen actions (familiarity: 4.82 +- 0.23; frequency of use: 3.88 +- 0.43) compared to workshop actions (familiarity: 3.62+-0.85; frequency of use: 1.77+-0.45), based on significant Wilcoxon rank sum tests (familiarity: z = 7.540, p < .001; frequency of use: z = 8.594, p < .001; Figure S1).

**Figure S1.** Familiarity and frequency of use of kitchen and workshop actions rated on a 5-point Likert-scale among a sample of psychology students at the University of Regensburg.

**Conclusion**

The findings suggest that bachelor students in psychology, at the University of Regensburg, are more familiar with kitchen actions than workshop actions, and perform kitchen actions more regularly than workshop actions, based on the set of actions used in the main study. These findings might reflect sample specific effects and might thus be different in students in other fields or might reflect general tendencies. Therefore, the results of this evaluation cannot be taken as evidence for general effects in broader populations.
